# Proteomics of Asrij Perturbation in *Drosophila* Lymph Glands for Identification of New Regulators of Hematopoiesis*[Fn FN2]

**DOI:** 10.1074/mcp.RA119.001299

**Published:** 2019-03-28

**Authors:** Saloni Sinha, Arindam Ray, Lakshman Abhilash, Manish Kumar, Sreelakshmi K. Sreenivasamurthy, T. S. Keshava Prasad, Maneesha S. Inamdar

**Affiliations:** From the ‡Jawaharlal Nehru Centre for Advanced Scientific Research, Jakkur, Bangalore 560064, India;; §Institute of Bioinformatics, International Technology Park, Bangalore 560066, India;; ¶Department of Immunology and Infectious Diseases, Harvard T.H. Chan School of Public Health, Boston, MA;; ‖NIMHANS-IOB Proteomics and Bioinformatics Laboratory, Neurobiology Research Centre, National Institute of Mental Health and Neurosciences, Bangalore 560029, India;; **Center for Systems Biology and Molecular Medicine, Yenepoya Research Center, Yenepoya (Deemed to be University), Mangalore-575018, India;; ‡‡Institute for Stem Cell Biology and Regenerative Medicine, GKVK, Bellary Road, Bangalore 560065, India

**Keywords:** Drosophila melanogaster*, Blood*, Differentiation*, iTRAQ, Knockouts*, Mass Spectrometry, Asrij, hematopoiesis, lymph gland, proteome

## Abstract

Hematopoiesis is the process of differentiation of precursor blood cells into mature blood cells that is controlled by a complex set of molecular interactions. Understanding hematopoiesis is important for the study of hematological disorders. However, a comprehensive understanding of how physiological and genetic mechanisms regulate blood cell precursor maintenance and differentiation is lacking. Owing to simplicity and ease of genetic analysis, the *Drosophila melanogaster* lymph gland (LG) is an excellent model to study hematopoiesis. Here, we quantitatively analyzed the LG proteome under genetic conditions that either maintain precursors or promote their differentiation *in vivo*, by perturbing expression of Asrij, a conserved endosomal regulator of hematopoiesis. Using iTRAQ-based quantitative proteomics, we determined the relative expression levels of proteins in Asrij-knockout and overexpressing LGs from 1500 larval dissections compared with wild type. Our data showed that at least 6.5% of the *Drosophila* proteome is expressed in wild type LGs. Of the 2133 proteins identified, 780 and 208 proteins were common to previously reported cardiac tube and hemolymph proteomes, respectively, resulting in the identification of 1238 proteins exclusive to the LG. Perturbation of Asrij levels led to differential expression of 619 proteins, of which 27% have human homologs implicated in various diseases. Proteins regulating metabolism, immune system, signal transduction and vesicle-mediated transport were significantly enriched. Immunostaining of representative candidates from the enriched categories and previous reports confirmed 73% of our results, indicating the validity of our LG proteome. Our study provides, for the first time, an *in vivo* proteomics resource for identifying novel regulators of hematopoiesis that will also be applicable to understanding vertebrate blood cell development.

## Abstract (precis)

Identification of molecules and processes that regulate hematopoiesis using *Drosophila* lymph gland (LG) as a model, is important for widening its scope and applicability as a tool to understand mechanisms regulating blood cell homeostasis. Using Asrij modulation, we compared the LG proteome under conditions that maintain precursors or promote differentiation *in vivo* and identified conserved as well as additional regulators of *Drosophila* hematopoiesis. The LG proteome provides an invaluable resource for studying insect as well as vertebrate blood cell development.

## Graphical Abstract

**Figure:**
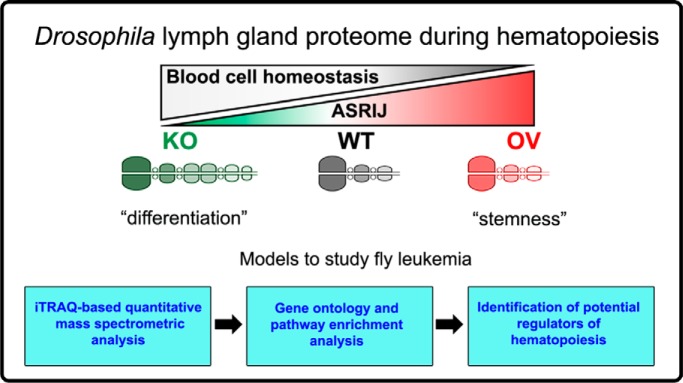

- First report on the quantitative proteomic profiling of *Drosophila* lymph glands.
- Comparative proteomic analysis under conditions of perturbed blood cell homeostasis.
- Resource for identifying new regulators of insect and vertebrate hematopoiesis.

Blood cell development (hematopoiesis) follows well-defined steps that are controlled by a complex set of molecular interactions in both invertebrates and vertebrates. Hematopoietic stem and progenitor cells (HSPCs)^1^ in *Drosophila* and vertebrates, give rise to an organized hierarchy of intermediates that eventually generate an array of terminally differentiated cells responsible for maintenance of the blood system (1). Differentiation of vertebrate HSPCs along each lineage is orchestrated by a team of transcription factors and signaling molecules. Owing to the high conservation of signaling pathways and proteins between *Drosophila* and vertebrate hematopoiesis (2), the *Drosophila* larval lymph gland (LG) is a relevant and well-accepted model for studying mechanisms underlying hematopoiesis (3).

The *Drosophila* third instar larval lymph gland (LG) lobes are composed entirely of blood cells and their precursors. They flank the cardiac tube and are interspersed by two pairs of pericardial cells (4). The anterior-most pair of lobes (primary lobes) are the most studied and have three major populations: differentiated blood cells (hemocytes) in the outer cortical zone (CZ), undifferentiated cells (pro-hemocytes) in the inner medullary zone (MZ) and a posterior signaling center (PSC) that functions as a stem cell niche to maintain hematopoiesis. The posterior lobes are poorly characterized but thought to comprise mainly of pro-hemocytes (3, 5). Although the LG tissue is believed to have limited cell lineage diversity, new subpopulations continue to be reported (6), however, the proteins expressed in these cells remain largely unknown. Further, systemic perturbations also affect blood stem cell maintenance and aberrant systemic signals can disrupt blood cell homeostasis (7, 8). Therefore, mapping the endogenous LG proteome is important to understand the hematopoietic niche, progenitor populations and blood stem cell maintenance, especially given its significance to vertebrate hematopoiesis. Although a proteomic investigation of the *Drosophila* LG is promising and likely to provide novel insights into the mechanisms governing blood cell homeostasis, it presents its own unique challenges. The microscopically small size coupled with the lack of automated dissection techniques have been major roadblocks that have prevented application of proteomics to the LG tissue.

In this study, we probed the *Drosophila* LG proteome under conditions that maintain stemness or promote differentiation *in vivo*, to identify potential regulators with hitherto unknown function in hematopoiesis. Earlier studies have established the role of Asrij as an important regulator of *Drosophila* hematopoiesis and immunity (7, 9, 10). Deficiency of Asrij leads to a situation mimicking fly leukemia characterized by hyperproliferation and increased differentiation of pro-hemocytes (10). Using the sensitized background of genetically modified *asrij* null mutant (knockout, KO) or overexpressing (OV) LGs, we report, for the first time, the peptide and protein compendium of the *Drosophila* larval LG, under conditions of normal as well as perturbed blood cell homeostasis. Our study provides a timely addition to the limited repertoire of LG proteins and informs about cellular processes and pathways critical for maintenance of blood cell homeostasis.

## EXPERIMENTAL PROCEDURES

### 

#### 

##### Fly Stocks

*Drosophila melanogaster* stocks were maintained as described before (10). *Canton-S* was used as the wild type reference strain. Based on the experimental design, *w1118* or appropriate GAL4 (*e33CGAL4*/*TM6tb* from K. Anderson) controls were also used. Other fly stocks used were *arj^9^/arj^9^* (Asrij knockout, KO) (10) and *UAS-Dmasrij* (7).

##### Experimental Design and Statistical Rationale

In this study, we aimed to perform a proteomic characterization of the *Drosophila melanogaster* lymph gland (LG). Owing to the limited amount of tissue available per LG, we chose to perform proteomic analysis using pooled samples. Pilot experiments conducted helped standardize the amount of protein that could be isolated from a given number of LGs. *Canton-S* was used as the wild type (WT) strain. To maximize identification of additional regulators of hematopoiesis, we probed the *in vivo* LG proteome under conditions that maintain blood cell progenitors or promote their differentiation, by modulating levels of Asrij (overexpression (OV) and knockout (KO)), an important regulator of *Drosophila* hematopoiesis (7, 10). Although technically demanding and challenging, we performed 1500 LG dissections from third instar *Drosophila* larvae for each genotype (WT, KO and OV) to obtain ∼300 μg of protein for performing iTRAQ-based quantitative proteomic analysis. Because of the small size of the LG and the immensely time-consuming process of dissection and isolation, doing biological replicates at the time at which these experiments were performed was not feasible. The lack of automated LG dissection protocols and the unique nature of the sample itself present unique and major challenges to collecting enough protein for the study. To overcome these roadblocks that have prevented application of proteomics to this sample, we chose to analyze hits obtained, by immunostaining, to validate our findings from the LG proteome.

Peptides generated by trypsin digestion from WT, KO and OV LGs were labeled with iTRAQ 4-plex reagents, as per manufacturer's protocol, yielding 114, 115, and 116 reporter ions, respectively. To increase coverage, iTRAQ-labeled peptides pooled from each genotype were split into 13 distinct fractions prior to LC-MS/MS analysis. Raw MS/MS data was processed using search engines Sequest and Mascot (version 2.4.1) in the Proteome Discoverer version 2.0 suite (Thermo Fisher Scientific) and results were exported as Microsoft Excel files (supplemental Tables S1 and S2) for further analysis. Peptide abundance values represented by iTRAQ reporter ion intensities were used to perform a Chi-square test to compare if the fold change of each peptide belonging to any two genotypes differs statistically from 1:1. We performed two tests for each peptide, vis-à-vis, (1) KO *versus* WT and (2) OV *versus* WT. Because of the large number of hypotheses being tested, we adjusted the *p* values of these tests using Benjamini-Hochberg (11) correction such that the net false discovery rate (FDR) is set at 1%. The relative expression of proteins was calculated based on the relative abundance for the corresponding unique peptides. For downstream analyses such as Gene Ontology and pathway enrichment, differentially abundant proteins were used, selection criteria for which included an adjusted *p* value <0.01 and fold change of <0.6 [based on Asrij (FBpp0305129) values] and >1.4. Although the lower limit of <0.6 was statistically derived, the upper limit of >1.4 was derived arbitrarily only to maintain symmetry in picking relevant regulated targets. Although experimental methods confirm the complete absence of Asrij in KO LGs (10), we obtained a KO/WT peptide abundance ratio of 0.59. This is most likely because of the iTRAQ-based quantitation approach adopted for our proteomics study, which is known to have issues with reporting reliable relative protein abundance estimates (12, 13). All the analyses described here were performed using custom scripts in R.

##### Drosophila melanogaster Lymph Gland (LG) Isolation for Proteomics Analysis

Wandering third instar larvae were immobilized by cooling, pinned ventral side up and a longitudinal excision was made. Viscera and excess parts of the body wall were removed; leaving a thin strip of body wall to which the dorsal vessel remained attached. The LG having the primary, secondary and tertiary lobes intact was collected in phosphate buffer saline (PBS) containing protease inhibitor mixture (Sigma) and phenylmethanesulfonylfluoride (Sigma) in order to prevent proteases from degrading the tissue. Dissected LGs were stored at −80 °C. 1500 LGs of desired genotype were lysed in 0.5% SDS, homogenized by sonication and centrifuged at 13,000 rpm for 10 min at 4 °C followed by protein estimation of the supernatants using bicinchoninic acid (BCA) assay (Thermo Fisher Scientific) for normalization on gel. Equivalent amounts of protein quantified spectrophotometrically from each sample was reduced and alkylated and then subjected to trypsin (Sequencing Grade Modified Trypsin, Promega) digestion in an enzyme to substrate ratio of 1:20 (w/w) at 37 °C for 16 h.

##### Mass Spectrometry Methodology

The pooled LGs were given to the proteomics mass spectrometry department of the Institute of Bioinformatics (IOB), Bangalore, for sample processing according to standard procedure. Peptides generated by trypsin digestion from WT, KO and OV LGs were labeled with iTRAQ 4-plex reagents (Applied Biosystems) as per manufacturer's protocol, yielding 114, 115, and 116 reporter ions, respectively. These iTRAQ-labeled peptides were eventually pooled, reconstituted in SCX solvent A (10 mm potassium phosphate, 20% acetonitrile, pH 2.8) and subjected to strong cation exchange chromatography on a polysulfoethyl A column (200 × 2.1 mm; 5 μm; 200 Å PolyLC, Columbia) using Agilent's 1200 series HPLC system. Fractionation of peptides was carried out by a linear gradient of solvent B (350 mm KCl in solvent A) for 70 min at a flow rate of 200 μl per minute. The fractions thus collected, were dried in a Speedvac, reconstituted in 10 μl of 0.1% TFA and cleaned using C_18_ stage tips prior to LC-MS/MS analysis.

Tandem mass spectrometric analysis of the iTRAQ-labeled peptides was carried out using LTQ-Orbitrap Velos mass spectrometer (Thermo Fisher Scientific) interfaced with Easy nanoLC II (Thermo Fisher Scientific). The nanospray ionization source of the mass spectrometer was fitted with a 10 μm emitter tip (New Objective) and maintained at 2000 V ion spray voltage. Peptide samples were loaded onto an enrichment column (2 cm × 75μ, Magic AQ C18 material 5μ particle size, 100 Å pore size) in 0.1% formic acid, 5% acetonitrile for 15 min and peptide separation was carried out on analytical column (10 cm × 75μ, Magic AQ C18 material 5μ particle size, 100 Å pore size) using a linear gradient of 7–35% solvent B (90% acetonitrile in 0.1% formic acid) for 60 min at a constant flow rate of 350 nl/minute. Data was acquired using Xcalibur 2.1 (Thermo Fisher Scientific) in a data-dependent manner in the *m*/*z* range of 350 to 1800 at a mass resolution of 60,000 at 400 *m*/*z* at the MS level and 15,000 at 400 m/z at MS/MS level by targeting the top 20 abundant ions for fragmentation using higher energy collisional dissociation at 39% normalized collision energy. The dynamic exclusion option was enabled during data acquisition with exclusion duration of 60 s. Lock mass option was enabled for real time calibration using polycyclodimethylsiloxane (*m*/*z*, 415.12) ions.

##### Database Search Parameters and Acceptance Criteria for Identifications

Raw MS/MS spectra files were searched against *Drosophila melanogaster* RefSeq protein database (release 70; 30,513 entries) appended with the known contaminants using SEQUEST and MASCOT (version 2.4.1) search engines in the Proteome Discoverer version 2.0 suite (Thermo Scientific, Germany). A precursor ion mass range of 600–5000 Da and a signal-to-noise ratio of 1.5 was used for the searches. Enzyme specificity was set to trypsin, allowing for a maximum of one missed cleavage. Variable (oxidation of methionine and phosphorylation of serine, threonine and tyrosine) and fixed (carbamidomethylation of cysteine; iTRAQ-labeling at N terminus of the peptide and lysine) modifications were selected. Mass tolerance was set to 15 ppm and 0.1 Da for precursor and fragment ions, respectively. Peptide lists were filtered to remove known contaminants such as BSA and human keratin proteins. To maximize the coverage of identifications, 1% FDR cut-off was used at PSM level for all the identifications as calculated by percolator algorithm using decoy search approach. Data analysis was performed using custom scripts in R.

##### Mass Spectrometry Data Analysis

Intensities of iTRAQ values from the MS/MS spectra were used to calculate peptide abundances using the 'peptide and protein quantifier' in Proteome Discoverer version 2.0 suite (Thermo Fisher Scientific). Peptide abundance scores were exported as Microsoft Excel file (supplemental Table S1) from the software to perform quantitative comparisons. FDR confidence for each protein was estimated and PSMs that did not qualify the 1% FDR were excluded from the analysis. Additionally, peptides shared between protein isoforms were excluded for quantitative estimation and only the unique peptides, identified across all LG genotypes, were used for the relative quantitation and statistical analyses, as described in Experimental Design and Statistical Rationale. Proteins that are discussed in the manuscript were manually inspected for the MS/MS spectra quality of the respective peptides.

##### In Silico Analysis

A web-based toolset g:Profiler was used for performing Gene Ontology (GO), pathway enrichment (PE) analysis and for identifying proteins with human homologs implicated in various diseases (https://biit.cs.ut.ee/gprofiler/) (14). Venn diagrams were made using the online tool Venny 2.1 (http://bioinfogp.cnb.csic.es/tools/venny/).

##### LG Immunostaining, Imaging and Analysis

To validate findings from the LG proteome, immunostaining was performed for selected proteins identified with multiple (at least 4) peptides with high confidence (supplemental Table S1). All the proteins selected for validation by LG immunostaining showed a good MS/MS spectra quality. Immunostaining analysis was performed for LGs isolated from KO and OV with appropriate controls (*Canton-S*, as the wild type control; *w1118*, as *asrij* mutation was made in this genetic background; and *e33CGal4*, as the parental control for OV) as described before (10). Images were captured with a Zeiss LSM880 confocal microscope. Primary antibodies used were against Rab7 (rabbit) and Rab11 (rabbit) (both from MarcosGonzalez Gaitan, University of Geneva); ARF1 (rabbit) (7); ATP5A (mouse), SDHB (mouse), CoxIV (mouse) and NDUFS3 (mouse) (all from Abcam). Secondary antibody was coupled to Alexa-Fluor 488 or 568 or 633 (all from Life Technologies). Estimation of area and fluorescence intensity of LG lobes was performed using Fiji (Image J) software for the primary, secondary and tertiary pair of LG lobes to analyze differences in protein expression across different genotypes. Statistical significance was estimated using two factor ANOVA (LG lobe and genotype being the two factors taken into consideration) followed by a post-hoc analysis in STATISTICA v5.0.

## RESULTS

### 

#### 

##### Mass Spectrometric Mapping of the Drosophila melanogaster Lymph Gland Proteome

Understanding the detailed molecular processes underlying *Drosophila* lymph gland (LG) hematopoiesis remains a challenge, despite the increasing attention it has received over the past few years. A proteomic analysis of the *Drosophila* LGs would reveal important additional clues and generate a resource for deeper understanding of hematopoiesis. However, the entire LG tissue is only about ∼1.5–2 mm in length, relatively transparent and made up of about ∼1000–1500 cells (15). This, coupled with a lack of technological developments, makes large scale microdissection of enough numbers of LGs for proteomic analysis extremely challenging. Owing to sampling issues, analysis thus far has been primarily genetic or performed using cultured S2 cells that represent embryonic hemocytes.

**Fig. 1.**
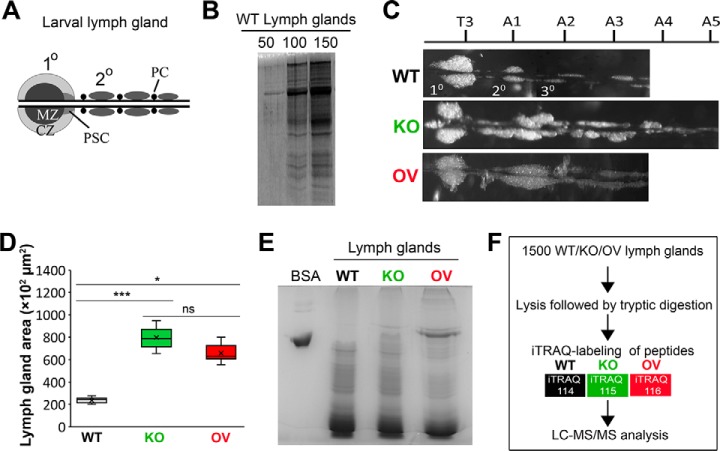
**Experimental design for mapping the proteome of *Drosophila melanogaster* lymph glands.** *A*, Schematic representation of a wild type (WT) lymph gland (LG). Primary (1^0^) and posterior (2^0^) LG lobes flank the cardiac tube and are interspersed by pericardial cells (PC). CZ: cortical zone; MZ: medullary zone; PSC: posterior signaling center. *B*, Protein quality verification by SDS-PAGE and Coomassie blue staining of lysates obtained from 50, 100 and 150 WT LGs. *C*, Representative images of WT, Asrij knockout (KO) and Asrij overexpressing (OV) LGs. Thoracic (T) and abdominal (A) segment numbers are indicated. Primary lobe is anterior to T3. *D*, Graph showing LG area across different genotypes (*n* = 10). *E*, Protein quality verification by SDS-PAGE and Coomassie blue staining of lysates obtained from 1500 WT, KO and OV LGs. BSA was used as positive control. *F*, Schematic representation of the protocol followed for comparative proteomic analysis of Asrij modified *Drosophila* LGs.

The *Drosophila* LG is heterogeneous and contains developmentally distinct zones (MZ, CZ, PSC) (Fig. 1*A*). Nevertheless, as compared with vertebrate bone marrow or *in vitro* cultured hematopoietic cells, it offers a relatively pure population of *in vivo* blood cells with limited cell lineage diversity. As cells are harvested from the natural context *i.e.* the *Drosophila* larva, this provides the added advantage of minimal artifact generation. Thus, we reasoned that although technically demanding and time consuming, manual dissection was imperative for direct sampling of LGs to obtain a reasonably good proteomic characterization of the *Drosophila* LGs. A detailed protocol for the isolation and collection of LG samples for proteomic analysis is described (see Experimental procedures).

The first and the most critical step toward deciphering the proteome of *Drosophila* LGs was performing large scale dissections for sample collection. As the amount of protein obtained from one LG is insufficient owing to its small size, it was necessary to pool LG samples for proteomic analysis. At the time at which this experiment was performed, logistical constraints compelled us to opt for a strategy wherein large-scale pooling of LG samples from a long-term inbred strain of *Drosophila* seemed feasible. We thought this to be appropriate for two reasons, *vis-a-vis*, (1) because of the inbred nature of our stocks, low among individual variation is less likely to yield erroneous expression values from the experiment (which could otherwise be dealt with by having multiple biological replicates), and as a consequence, (2) inference regarding expression levels of proteins could be made with greater confidence as the values are more likely to represent the population level expression value. For deciding upon the number of LG samples to be pooled, we standardized and evaluated the amount of protein that could be extracted from a given number of LGs. Protein concentrations of lysates prepared from 50, 100, and 150 wild type (WT, *Canton-S*) LGs were estimated and the corresponding protein profiles were examined using SDS-PAGE followed by Coomassie Blue staining (Fig. 1*B*). Our results suggested that ∼30 μg protein could be isolated by dissecting 150 WT LGs and hence we estimated that dissecting 1500 LGs should yield enough protein (∼ 300 μg) for performing a successful proteomics experiment.

To increase the prospect of identifying novel regulators of hematopoiesis, we chose to inspect the proteome of *asrij* null mutant (“knockout,” KO) and overexpressing (OV) LGs, which mostly represent the differentiated and undifferentiated blood cell states, respectively (7, 10, 16). Compared with control, KO LGs show premature differentiation, resulting in increased numbers of plasmatocytes and crystal cells (10), whereas OV LGs do not show aberrant differentiation and can maintain blood cell homeostasis (16). Although there is no gross difference in morphology at the embryonic, first and second instar stages, by the third instar stage, KO LGs develop increased number of posterior lobes, which are asymmetric and extend up to abdominal segments A4 or A5 along with a disrupted pericardial cell arrangement (10) (Fig. 1*C*). When quantified, both KO and OV LGs show significantly increased area as compared with WT (Fig. 1*D*), owing to the increased sizes of the secondary and tertiary lobes (supplemental Fig. S1*A*–S1*C*). Based on these characteristics of the *asrij* mutants, we reasoned that performing a comparative analysis of LGs harvested and pooled, from each of the three different genotypes- WT, KO and OV (inbred *Drosophila* strains), might make it easier to find the major proteome changes accompanying hematopoiesis. Hence, 1500 LGs from staged wandering third instar *Drosophila* larvae were manually dissected, pooled and total protein was extracted (see Experimental Procedures). The lysates obtained included proteins from the primary lobes, posterior lobes, two pairs of pericardial cells and the cardiac tube (Fig. 1*E*). Subsequently, the peptides isolated from WT, KO and OV LGs were differentially labeled with iTRAQ 4-plex reagents, subjected to quantitative mass spectrometry and analyzed for the effect of Asrij deletion or overexpression for each pooled sample (see Experimental Procedures, Fig. 1*F*).

##### Overview of the Drosophila melanogaster Lymph Gland Proteome

**Fig. 2.**
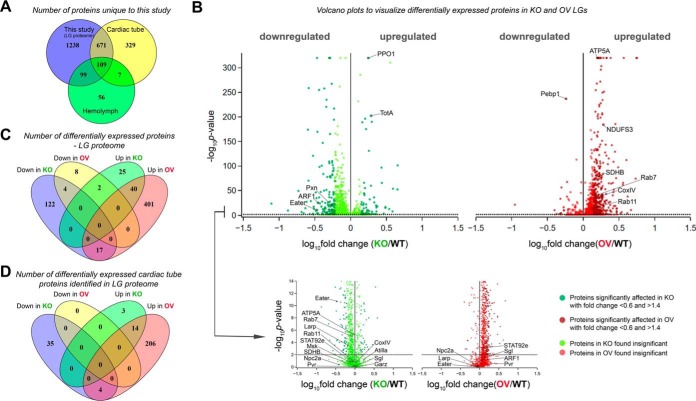
**Proteomic analysis of *Drosophila melanogaster* lymph glands.** *A*, Venn diagram representing the distribution of proteins in WT LG samples identified in our study and previously reported studies (17, 18). *B*, Representation of all identified proteins in KO and OV LGs by volcano plot. Dotted black line represents the *p* value cut-off used. Darker shade of color in each of the volcano plots represents the proteins whose expression is significantly affected. *C*, Venn diagram representing number of upregulated and downregulated differentially expressed (DE) proteins in KO and OV LGs. *D*, Venn diagram representing number of cardiac tube proteins DE in KO and OV LGs.

Searches of the mass spectrometry derived data against the *Drosophila melanogaster* RefSeq protein database (release 70) using Proteome Discoverer software (version 2.0) identified 2133 LG proteins, supported by more than 9900 peptides with a total of 23140 peptide spectral matches (PSMs) (supplemental Table S2). This indicates that at least 6.5% of the *Drosophila* proteome is expressed in the third instar *Drosophila* larval LG. To assess the tissue specificity of our LG proteome, we compared our dataset to the already reported proteomic profiles of the cardiac tube (17) and hemolymph (18). Of the 2133 proteins identified, 780 have been previously reported to express in the adult fly cardiac cells (17) and 208 in larval hemolymph (18) (see Fig. 2*A* and supplemental Table S3). Although no proteomic study of pericardial cells has been reported till date, an *in vivo* functional analysis study reported 80 genes to be expressed in pericardial nephrocytes (19). No common proteins were found upon comparison of the data sets, probably owing to the underrepresentation of pericardial cells (4–6 cells/LG). This indicates that a bulk of the 1238 proteins are newly identified and have not been reported earlier in the LG (Fig. 2*A*). Most of these identified proteins are likely to be expressed exclusively in the LG lobes.

Several proteins with well-defined roles in the regulation of *Drosophila* blood cell homeostasis were identified in our proteome. Known regulators of blood cell survival, proliferation and differentiation such as Eater (FBgn0243514), ADP-ribosylation factor 1 (ARF1, FBgn0010348), signal transducer and activator of transcription protein at 92E (STAT92e, FBgn0016917), gartenzwerg (Garz, FBgn0264560), PDGF- and VEGF-receptor related (Pvr, FBgn0032006), Niemann-Pick type C 2a (Npc2a, FBgn0031381), La related protein (Larp, FBgn0261618), Moleskin (Msk, FBgn0026252), Prophenoloxidase 1 (PPO1, FBgn0283437), Atilla (FBgn0032422) and Peroxidasin (Pxn, FBgn0011828) were identified. Additionally, proteins involved in regulation of immunity [Turandot A (TotA, FBgn0028396), Phosphatidylethanolamine Binding Protein 1 (Pebp1, FBgn0038973)] and LG development (Sugarless (Sgl, FBgn0261445)) were also identified (Fig. 2*B*, supplemental Table S2), thus validating our approach. Taken together, comparison with existing proteome datasets of cardiac tube cells and hemolymph and identification of known regulators of LG hematopoiesis and development, demonstrates that our approach has successfully yielded a LG-enriched proteome.

##### Identification of the Drosophila Lymph Gland Proteome Responsive to Asrij

To identify proteins showing differential expression, we compared the abundance ratios of peptides detected in Asrij modulated conditions, across all three LG genotypes (WT, KO and OV). Although KO LGs show complete absence of transcript and protein expression of *asrij* (10), the proteomic analysis showed a KO/WT ratio of 0.59 for Asrij. This quantitation was based on the one unique peptide (FBpp0305129) identified against Asrij. This is likely because of the interference of mixed MS/MS events from isobaric peptides that occur during precursor selection and can lead to underestimation of quantitative differences (12, 13). Based on statistical analyses and the peptide abundance ratio of Asrij in KO/WT, proteins with a fold change <0.6 or >1.4 and an adjusted *p* value <0.01, were identified as differentially expressed (see Experimental Procedures and supplemental Table S4). For visual representation of these differentially expressed proteins, volcano plots were generated (Fig. 2*B*). Expression of 619 proteins significantly changed as compared with WT and changes observed in the proteome profile were mostly synergistic with Asrij levels. As compared with WT, KO showed reduced expression of 143 out of 210 proteins, whereas 458 out of 472 proteins were overexpressed in Asrij OV (Fig. 2*C*). Of these, 17 proteins were proportionately regulated by Asrij, *i.e.* down in KO and up in OV, whereas 2 proteins showed opposite changes in abundance when compared with Asrij levels (Fig. 2*C*). Thus, the LG proteome is sensitive to Asrij levels. Interestingly, of the 780 cardiac tube proteins identified in the LG proteome, 262 were significantly affected (56 in KO (39 downregulated and 17 upregulated) and 224 in OV (all upregulated)), suggesting that Asrij may be involved in playing a role in remodeling the cardiac tube tissue to facilitate stromal interactions on *Drosophila* hematopoietic development (Fig. 2*D*, supplemental Table S5). Among the 619 differentially expressed proteins, human homologs of 166 proteins were found to be implicated in various diseases such as “abnormality of metabolism/homeostasis” (HP:0001939, *p* = 8.3E-05), “respiratory insufficiency” (HP:0002093, *p* = 0.0387), and “abnormality of the mitochondrion” (HP:0003287, *p* = 1.67E-08), among others (supplemental Table S6). The novel proteins identified from our study can now be targeted to generate *Drosophila* models for a wide variety of hematopoietic as well as metabolic disorders.

##### Functional Annotation Enrichment Analysis

**Fig. 3.**
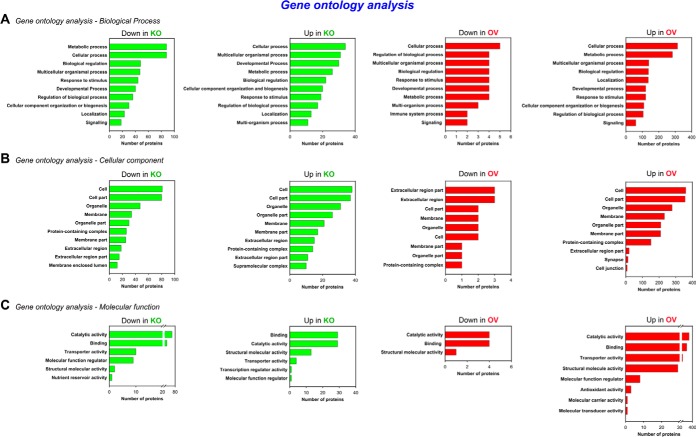
**Functional enrichment analysis.** Bar plots representing Gene Ontology analysis of the differentially expressed proteins based on (*A*) biological process, (*B*) cellular component and (*C*) molecular function, done using the g:Profiler classification. The *x* axis shows the number of proteins in each category.

To define how the Asrij-regulated proteome affects hematopoiesis, Gene Ontology (GO) analysis of the 619 differentially expressed proteins was performed using tools available from g:Profiler (14) to categorize proteins according to their biological function, cellular component and molecular function (Fig. 3). The biological processes mediated by Asrij mainly included “metabolic processes” (GO: 0008152), “cellular processes” (GO: 0009987), “multicellular organismal process” (GO: 0032501), among others (Fig. 3*A*). Enrichment of “metabolic processes” is not surprising given the already established role of metabolism in regulation of stem cell fate (20) and the ability of Asrij to regulate energy metabolism in human pluripotent stem cells (21). Further, “cell communication” (GO: 0007154) and “cell cycle” (GO: 0007049) were the major sub-categories enriched in “cellular processes.” The cellular components involved encompassed “cell” (GO: 0005623), “cell part” (GO: 0044464), “organelle” (GO: 0043226), “organelle part” (GO: 0044422), “extracellular region” (GO: 0005576), etc. (Fig. 3*B*). Molecular functions enriched for Asrij were primarily related to “binding” (GO: 0005488), “catalytic activity” (GO: 0003824), “structural molecular activity” (GO: 0005198), etc. (Fig. 3*C*).

**Fig. 4.**
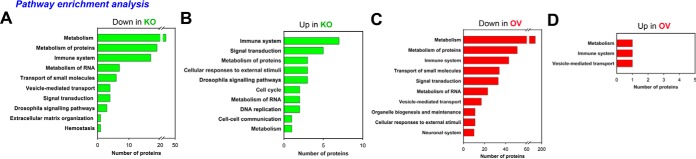
**Pathway enrichment analysis.** *A–D*, Pathway enrichment analysis of the differentially expressed proteins performed using the g:Profiler (Biological Pathways: Reactome) classification. The *x* axis shows the number of proteins in each category.

Pathway enrichment (PE) analysis of the 210 (143 downregulated, 67 upregulated) and 472 (14 downregulated, 458 upregulated) proteins perturbed in KO and OV LGs, respectively, performed using g:Profiler (Biological Pathways: Reactome) (14) revealed a significant enrichment of protein clusters involved in regulation of metabolism (R-DME-1430728), immune system (R-DME-168256), transport of small molecules (R-DME-382551), vesicle-mediated transport (R-DME-5653656) and signal transduction (R-DME-162582), among others (Fig. 4*A*–4*D*). As Asrij plays an important role in regulating diverse cellular processes such as mitochondrial oxidative phosphorylation (21), immunity (9) and endocytosis (7, 10), enrichment of the above-mentioned pathways in the Asrij perturbed (KO and OV) LG proteomes is expected and consistent with known functions of Asrij (7, 9, 10, 21).

##### Validation of Candidates Identified from the Drosophila Lymph Gland Proteome

As it was not practical to perform biological replicates owing to the unique challenges associated with sample collection, we validated the proteome in two ways: (1) by comparing changes in protein levels assessed by the proteome to that expected, based on the known function and mechanism of action of Asrij, as per reports from the literature (7, 10, 16), and (2) by analyzing protein expression of representative candidates by immunostaining LGs. To understand the effect of *asrij* dosage on perturbed expression of the candidate proteins, validation by immunostaining was performed using the WT (*Canton-S*) control and the relevant genetic background controls for KO (*w1118*) and OV (*e33cGAL4*) (see Experimental procedures).

**Fig. 5.**
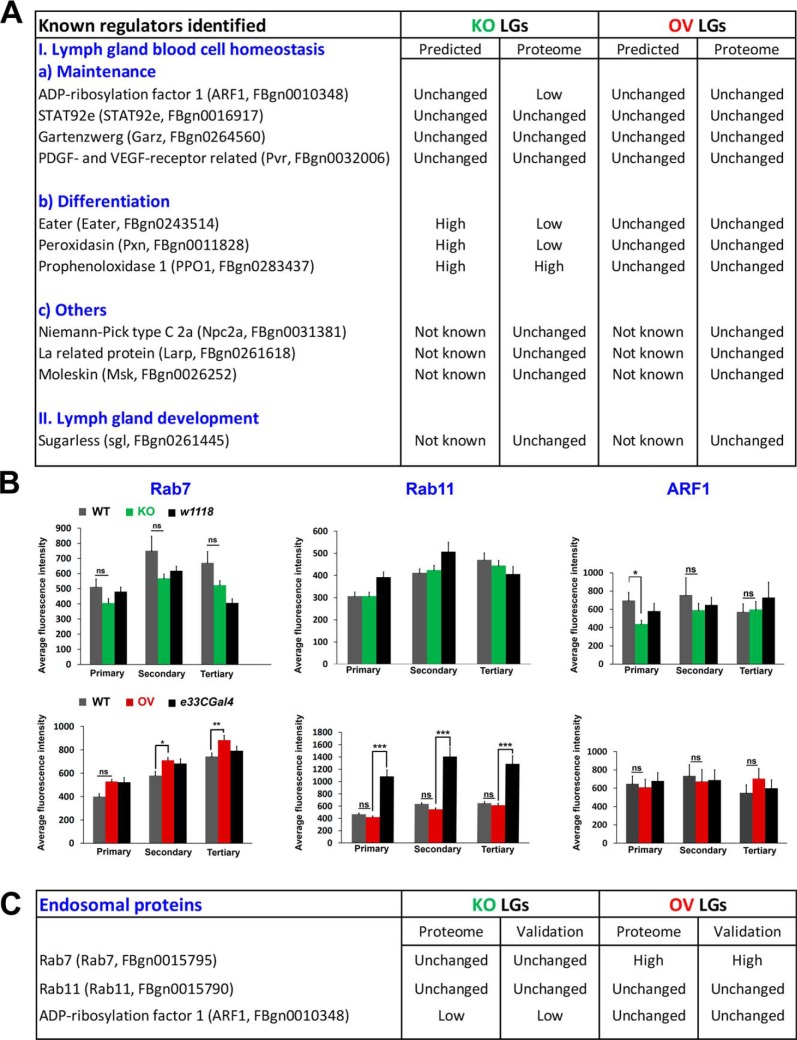
**Validation of endosomal hits Rab7, Rab11 and ARF1 obtained from the LG proteome.** *A*, Comparison of predicted and proteome-obtained expression levels of known regulators of *Drosophila* LG blood cell homeostasis and development. *B*, Graphs showing average fluorescence intensity levels of Rab7, Rab11 and ARF1 across primary, secondary and tertiary LG lobes. Genotypes are as indicated. Error bars represent standard error of mean and 'ns' indicates statistically non-significant difference. * *p* < 0.05, ** *p* < 0.01. *C*, Comparison of proteome-obtained and experimentally validated expression levels of LG Rab7, Rab11, and ARF1.

Asrij depletion does not affect ARF1 levels in circulatory hemocytes (7), however, the LG proteome showed ARF1 as reduced in KO and unchanged in OV (Fig. 5*A*). Validation by immunostaining (see below) showed that this was indeed the case, wherein we observed significantly reduced levels of ARF1 in the primary lobes of KO LGs (Fig. 5*B*, *C* and supplemental Fig. S2). The inconsistency between the predicted (unchanged expression, based on circulatory hemocyte data) and the obtained/validated (low expression, based on LG proteome) expression of ARF1 in LG is likely because of the different cell populations being compared. Unlike circulatory hemocytes, which comprise differentiated blood cells in majority, the LG is a more heterogeneous population that includes progenitors, differentiated blood cells and niche cells. Like ARF1, levels of Garz and STAT92e are not expected to change, based on previous reports that show Asrij affects their activation, but not total levels (7, 16). The same holds true for Pvr, which was shown to act upstream of Asrij (7), hence not expected to change in levels. In agreement with this, the proteome data shows that Garz, Stat92e and Pvr levels are unchanged in both KO and OV LGs (Fig. 5*A*). As Asrij KO LGs have increased differentiation to plasmatocytes and crystal cells, their respective markers, Eater (for plasmatocytes), Pxn and PPO1 (for crystal cells) could be expected at high levels in the KO proteome and likely unchanged in the OV LGs. Although KO LGs showed significantly increased PPO1 expression, matching our expectation, both Eater and Pxn levels were low in the KO proteome, though unchanged in OV (Fig. 5*A*). The relation of Asrij to other identified regulators of blood cell homeostasis (Npc2a, Larp, Msk) and LG hematopoiesis (sgl) is not known (Fig. 5*A*). Thus, the change in level of 5/7 proteins in KO and 7/7 proteins in OV LG proteome matched with that expected/reported.

To further strengthen the applicability of the LG proteome, we analyzed protein expression of representative candidates by immunostaining. Based on results obtained from PE analysis (Fig. 4*A*–4*D*) and the reported role of Asrij (7, 10, 21), we selected proteins belonging to the categories transport of small molecules (R-DME-382551), vesicle-mediated transport (R- DME-5653656) and metabolism (R-DME-1430728) for experimental validation. Given the proven role of Asrij in the endosomal trafficking pathway (7), we validated levels of proteins involved in mediating vesicle-mediated transport and transport of small molecules (Rab7, Rab11 and ARF1) by immunostaining LGs with the respective antibodies. The proteome data indicated Rab7 and Rab11 levels are not affected in KO LG, whereas the known Asrij interactor, ARF1 (7), is significantly low. Conversely, Rab7 levels are significantly high upon Asrij overexpression, whereas ARF1 and Rab11 are unchanged. Validation of these data by immunofluorescence-based analysis (Fig. 5*B* and supplemental Fig. S2*A*–S2*C*), showed that protein levels for all three endosomal molecules were as per the proteome analysis (Fig. 5*C*).

**Fig. 6.**
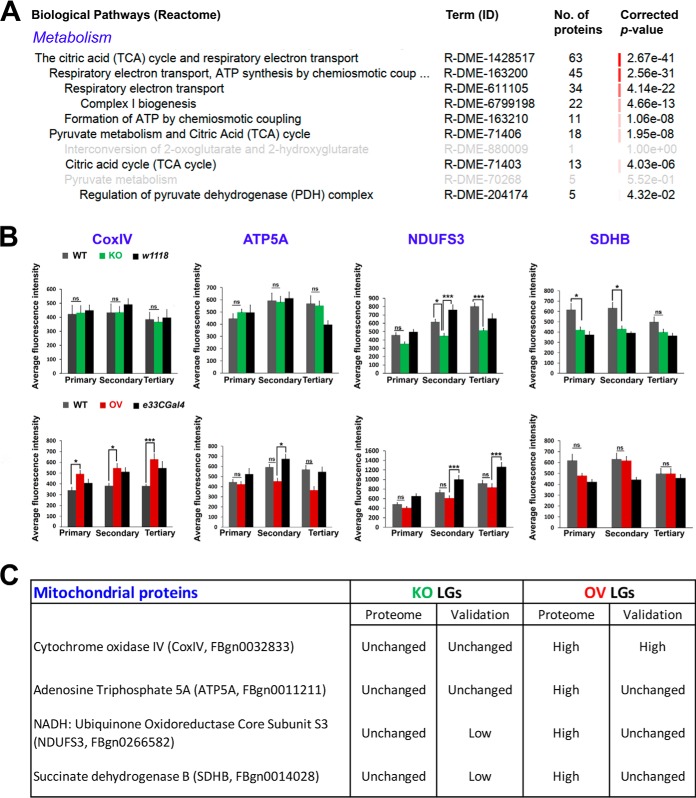
**Validation of mitochondrial hits CoxIV, ATP5A, NDUFS3 and SDHB obtained from the LG proteome.** *A*, Sub-categories significantly enriched under the category “metabolism” in Asrij OV LGs. *B*, Graphs showing average fluorescence intensity levels of CoxIV, ATP5A, NDUFS3 and SDHB across primary, secondary and tertiary LG lobes. Genotypes are as indicated (n>7 per genotype). Error bars represent standard error of mean and “ns” indicates statistically non-significant difference. * *p* < 0.05, ** *p* < 0.01 and *** *p* < 0.001. *C*, Comparison of proteome-obtained and experimentally validated expression levels of LG CoxIV, ATP5A, NDUFS3 and SDHB.

In human embryonic stem cells, Asrij/OCIAD1 regulates mitochondrial energy metabolism and interacts with components of the electron transport chain (21). Because energy metabolism (sub-categories: TCA cycle (R-DME-1428517), respiratory electron transport (R-DME-611105), complex I biogenesis (R-DME-6799198)) was a major perturbed category (Fig. 6*A*), we tested expression of mitochondrial molecules such as COXIV, ATP5A, NDUFS3 and SDHB, whose levels were unchanged in KO and significantly upregulated in OV, as per the LG proteome. Immunostaining of KO and control LGs with the respective antibodies showed that although COXIV and ATP5A levels were unchanged, NDUFS3 and SDHB levels were significantly downregulated in KO LGs as compared with *Canton-S* (Fig. 6*B* and supplemental Fig. S3*A*–S3*D*). The OV LGs showed significantly increased COXIV levels, unchanged ATP5A, NDUFS3 and SDHB levels, as compared with *Canton-S* (Fig. 6*B* and supplemental Fig. S3*A*–S3*D*). Based on results obtained from LG immunostaining, change in level of 2/4 proteins in KO and 1/4 proteins in OV agreed with the proteome data (Fig. 6*C*).

Thus, combining these two approaches, we find that levels of 9/13 proteins in KO and 10/13 in OV shown by the proteome are valid, giving high confidence to our analysis. These data indicate that our comparative proteome analysis is quite reliable and can be used as a resource for further studies.

## DISCUSSION

Studying hematopoiesis in *Drosophila* is far simpler than in vertebrates owing to the limited gene redundancy and few blood cell lineages. Although this makes analysis of gene function relatively easier in *Drosophila*, understanding how proteins and their signaling networks regulate hematopoiesis remains challenging. Proteomic analysis using genetically modified *Drosophila* LGs allowed us to identify potential regulators of hematopoiesis, which are relevant *in vivo* and whose active regulatory role would otherwise be masked. Here, for the first time, we present a detailed view of the *Drosophila* LG proteome under conditions that maintain blood cell precursors or trigger their aberrant differentiation using Asrij overexpressing and *asrij* null LGs, respectively, as models. In this analysis, we could identify at least 15.3% of the total protein-coding genes annotated in the latest release of FlyBase (annotation release 6.25). Also, identification of most of the proteins reported earlier in the cardiac tube and hemolymph, in our study, supports the LG proteome.

Changes in expression levels of most of the known regulators of blood cell survival, proliferation and differentiation, upon Asrij modulation, agree with earlier reports. For example, whereas the KO LG proteome showed a significant increase in PPO1 expression, no change was observed in Atilla expression in KO/OV LGs, which is expected and agrees with previously published data (9, 10). However, the increased expression of TotA, a downstream effector of the JAK/STAT pathway, observed in the KO LG proteome is surprising as Asrij depletion results in decreased activation of STAT92e (16). These findings coupled with the *in vivo* immunofluorescence based validation of candidate proteins boost confidence in the LG proteome. Validation of the LG proteome data involved comparing the expression of candidate proteins in KO and OV LGs to the WT control (*Canton-S*) and the relevant strain background controls (*w1118*, *e33CGAL4*) to accurately identify the effect of dosage of *asrij* on candidate protein expression. Inclusion of *Canton-S* as a control was necessary to test the quality and reliability of the LG proteome, as this was the only control used during mass spectrometry. As various parameters differ between a wild type and a strain background control, which can be attributed to differential genetic constitution and activity (22, 23), it was also important to consider *w1118* and *e33CGAL4* as experimental controls. Our findings show that a majority of endosomal and mitochondrial hits agree with the LG proteome data (5/7 in KO and 4/7 in OV), further increasing its reliability and applicability.

Our data support the idea that endosomal proteins can effectively modulate the net output of various other cellular processes such as oxidative phosphorylation and metabolism; and highlights the ability of the “endosomal matrix” (24) to modulate a wide range of targets in a context-specific manner. Moreover, identification of other molecules involved in mediating vesicle-mediated transport and endocytosis from the LG proteome, warrants further investigation of these pathways in maintaining blood cell homeostasis. Thus, Asrij can promote specific signaling outcomes from multiple signals that intersect to maintain blood cell homeostasis.

Among biological processes, the largest impact of Asrij perturbation in LGs was on proteins involved in metabolism. In vertebrates, although hematopoietic stem cells (HSCs) derive energy primarily from glycolysis, differentiated blood cells utilize oxidative phosphorylation (25). Also, the metabolic state plays an important role in determining HSC fate (25). Deregulation of the metabolic machinery in HSCs has been reported to result in leukemia (26, 27). Recently we showed that depletion of OCIAD1, the human ortholog of Asrij, causes enhancement of electron transport chain complex-I activity leading to increased differentiation of human embryonic stem cells to early mesodermal progenitors, which are the precursors of HSCs (21).We propose that Asrij might be involved in regulating important metabolic functions including regulation of oxidative phosphorylation machinery during hematopoiesis. Interestingly, our *in vivo* validation shows that although LG COXIV, NDUFS3 and SDHB levels are sensitive to Asrij levels, ATP5A is not. The difference in ATP5A levels observed in the LG proteome could also be because of significant contribution from the cardiac tube, which is energy dependent, based on the mitochondrial electron transport chain. The role of these molecules in hematopoiesis can now be tested in insect models like *Drosophila* and vertebrate models like mouse.

Perturbing Asrij levels affects mitochondrial morphology in hESCs (21). Interestingly, the LG proteome reveals that a key regulator of mitochondrial dynamics, Dynamin related protein 1 (Drp1, FBpp0077424), is significantly perturbed, in direct proportion to Asrij levels. The role of mitochondrial dynamics in hematopoiesis is a relatively underexplored subject. Recent reports suggest an essential role for regulators of mitochondrial dynamics in lymphoid lineage specification (28) and HSC self-renewal (29). It would be interesting to test how components regulating mitochondrial dynamics affect maintenance and differentiation of blood progenitors to various lineages in *Drosophila* as well as vertebrates.

In addition to the cardiac tube proteins, several proteins involved in muscle development were affected in Asrij mutant LGs. For example, proteins such as dystroglycan (FBpp0297348), an important structural constituent of muscle; and activity-regulated cytoskeleton associated protein 1 (Arc1, FBpp0086687) were significantly downregulated in KO, whereas, tropomyosin 2 (FBpp0291171), upheld (FBpp0073682) and myosin alkali light chain 1 (FBpp0088688) were significantly upregulated in OV LGs. The possible function of the above-mentioned proteins in regulation of LG hematopoiesis is intriguing. Alternatively, the LG could generate systemic signals that regulate cardiac muscle gene expression.

Perturbation of the hemocyte-specific protein Asrij triggers substantial remodelling of the LG proteome that could serve as a resource to unravel protein networks and circuitry that control human hematopoiesis. Further, Asrij/OCIAD1 in humans is associated with several carcinomas and imparts resistance to radiotherapy and chemotherapeutic drugs such as paclitaxel (30). An extensive study on various aspects of the LG proteome in invertebrate as well as vertebrate models may aid in unraveling new candidates, possibly with a pivotal role in regulating human hematopoiesis.

## DATA AVAILABILITY

The raw mass spectrometry data has been submitted to the ProteomeXchange Consortium (http://proteomecentral.proteomexchange.org) via the PRIDE (https://www.ebi.ac.uk/pride/archive/) (31) partner repository with the dataset identifier PXD010753. The data has also been submitted to MassIVE (https://massive.ucsd.edu/) and is available under the accession MSV000079315 or for direct download through ftp://MSV000079315@massive.ucsd.edu.

## Supplementary Material

Inamdar_Supplementary Table S1

Inamdar_Supplementary Table S2

Inamdar_Supplementary Table S3

Inamdar_Supplementary Table S4

Inamdar_Supplementary Table S5

Inamdar_Supplementary Table S6

Inamdar_Supplementary Text

## Footnotes

^1^ The abbreviations used are:

HSPC: hematopoietic stem and progenitor cell
ARF1: adenosine diphosphate (ADP)-ribosylation factor 1
ATP5A: adenosine triphosphate (ATP) synthase subunit alpha
BCA: bicinchoninic acid
BSA: bovine serum albumin
CoxIV: cytochrome oxidase subunit IV
DAPI: 4′,6-diamidino-2-phenylindole
Drp1: dynamin related protein 1
FDR: false discovery rate
GO: gene ontology
hESC: human embryonic stem cell
iTRAQ: isobaric tags for relative and absolute quantitation
KO: knockout
Larp: la related protein
LG: lymph gland
MassIVE: mass spectrometry interactive virtual environment
Msk: moleskin
NCBI: national center for biotechnology information
NDUFS3: NADH ubiquinone oxidoreductase core subunit S3
Npc2a: niemann-pick type C 2a
OV: overexpressing
PBS: phosphate buffer saline
PE: pathway enrichment
PSM: peptide-spectrum match
Rab: ras related GTP binding protein
SDHB: succinate dehydrogenase subunit B
SDS-PAGE: sodium dodecyl sulfate-polyacrylamide gel electrophoresis
TCA cycle: tricarboxylic acid cycle.
