# Supplementary material for: Proteomics of Asrij Perturbation in Drosophila Lymph Glands for Identification of New Regulators of Hematopoiesis
Source: Mol Cell Proteomics. 2019 Mar 28;18(6):1171–82. doi: 10.1074/mcp.RA119.001299 (PMC6553936; doi:10.1074/mcp.RA119.001299)
Supplement: Inamdar_Supplementary Text [file 142755_1_supp_297320_pv0cbm.pdf]

Proteomics of Asrij perturbed *Drosophila* lymph glands as a resource for identification of new regulators of hematopoiesis.

Saloni Sinha[1], Arindam Ray[1], Lakshman Abhilash[1], Manish Kumar[2,3], Sreelakshmi K. Sreenivasamurthy[2,4], T. S. Keshava Prasad[2,5] and Maneesha S. Inamdar[1,6*]

Inventory of Supplementary Information

1. Supplementary Figures and Figure Legends
2. Supplementary Table Legends

Supplementary Figures and Figure Legends

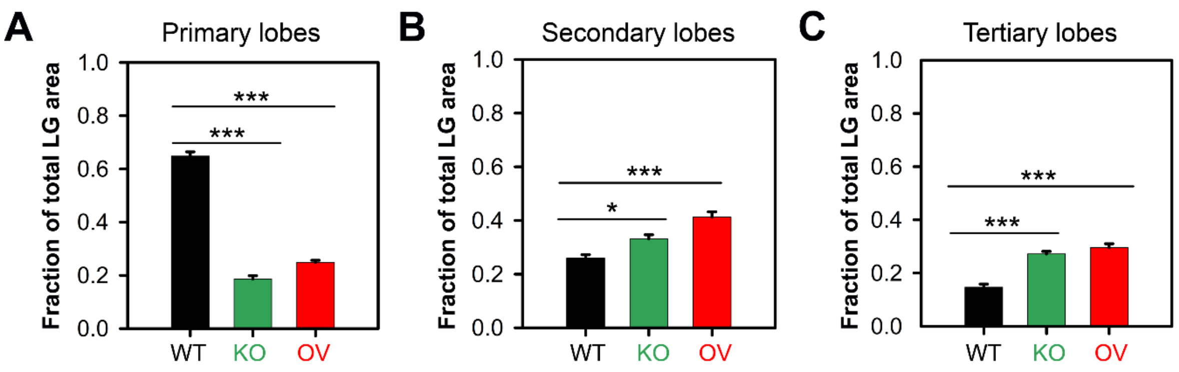

Supplementary Figure S1 (related to Figure 1). Graphs showing area of (A) primary, (B) secondary and (C) tertiary lobes as a fraction of the total LG area of WT, KO and OV. Error bars represent S.E.M. * p<0.05 and ***p<0.001.

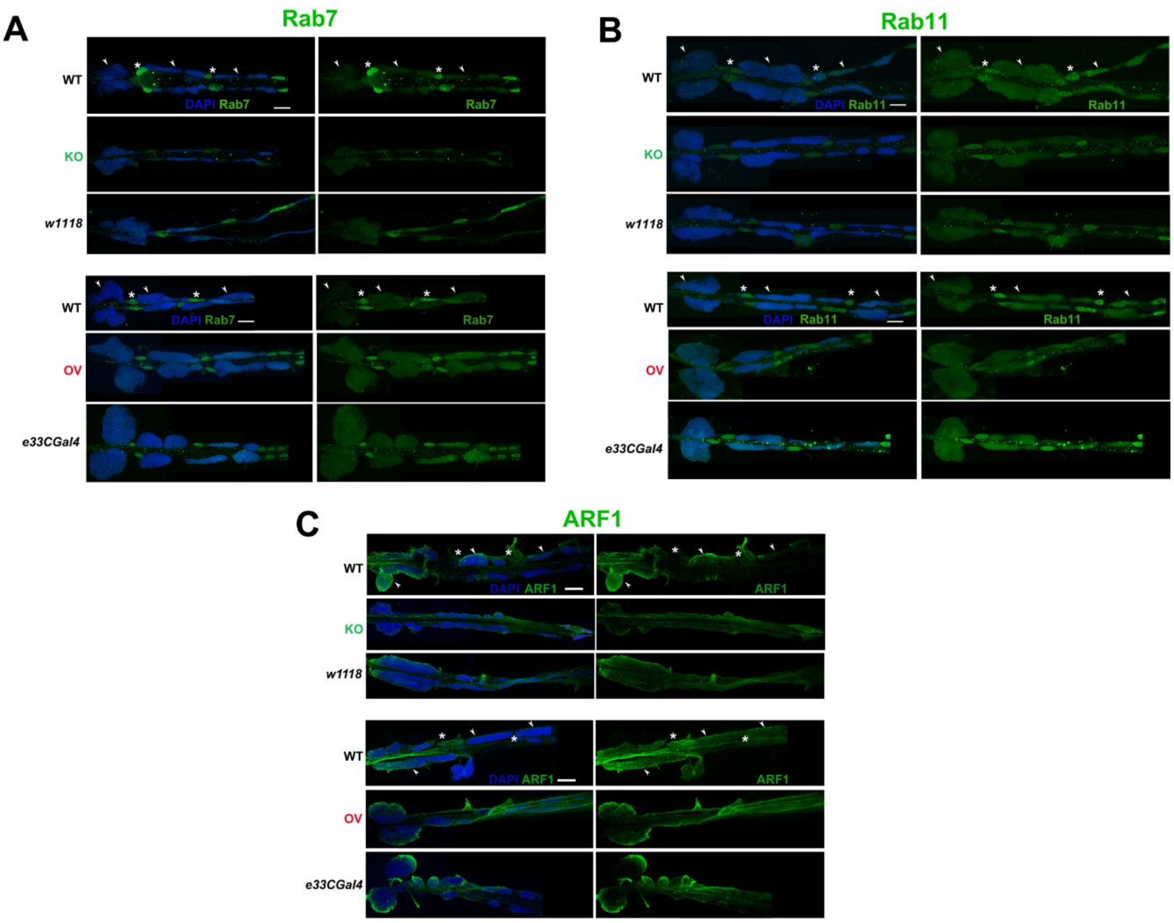

Supplementary Figure S2 (related to Figure 5). Immunofluorescence based validation of endosomal hits identified from the LG proteome. Third instar LG whole mounts showing expression of endosomal proteins **(A)** Rab7, **(B)** Rab11 and **(C)** ARF1 in different genotypes as indicated (n>7 per genotype). Nuclei were stained with DAPI (blue). Arrowheads and asterisks in the LG mark the lobes and the pericardial cells, respectively. Scale bar: 100 µm.

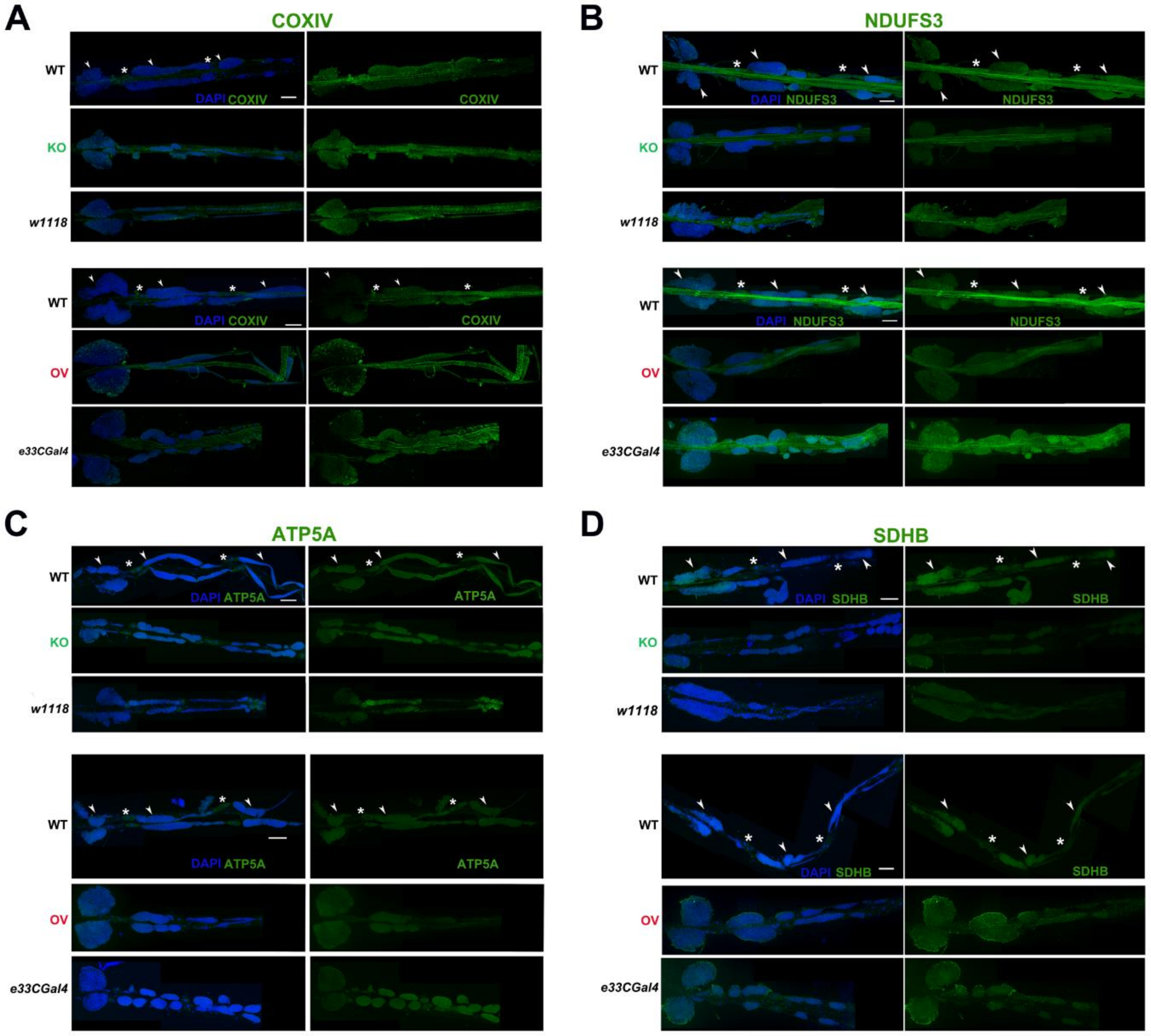

Supplementary Figure S3 (related to Figure 6). Immunofluorescence based validation of mitochondrial hits identified from the LG proteome. Third instar LG whole mounts showing expression of mitochondrial proteins **(A)** CoxIV, **(B)** NDUFS3, **(C)** ATP5A and **(D)** SDHB in

different genotypes as indicated (n>7 per genotype). Nuclei were stained with DAPI (blue). Arrowheads and asterisks in the LG mark the lobes and the pericardial cells, respectively. Scale bar: 100 µm.

Supplementary Table Legends

Table S1. List of peptides generated from the quantitative LG proteomic analysis.

Table S2. List of proteins identified in the LG proteomic analysis and their respective fold-change values upon Asrij KO and OV.

Table S3. Comparison of proteins obtained in the LG proteome with the cardiac and hemolymph proteomes. Lists of proteins identified in the LG proteome (our study) and of proteins detected in the cardiac tube (Cammarato et al., 2011), hemolymph (Handke et al., 2013) and pericardial cells (Zhang et al., 2013).

Table S4. List of differentially expressed LG proteins upon Asrij perturbation. "Up" and "down" indicate upregulation or downregulation of protein expression levels, respectively.

Table S5. List of cardiac tube proteins differentially expressed in KO and OV LGs.

Table S6. List of differentially expressed LG proteins with human homologs implicated in diseases.
